# Hemorrhagic Transformation Assessment Based on Dual Energy CT of Immediately and Twenty-Four Hours after Endovascular Thrombectomy for Acute Ischemic Stroke

**DOI:** 10.3390/diagnostics13152493

**Published:** 2023-07-27

**Authors:** Tianyu Wang, Weili Ding, Qing Chen, Zhongxiang Ding

**Affiliations:** 1 Department of Radiology, Affiliated Hangzhou First People’s Hospital, Zhejiang University School of Medicine, Hangzhou 310006, China; tianyuwang1221@zju.edu.cn; 2 Graduate School of Zhejiang Chinese Medical University, Hangzhou 310053, China; weili15825792618@163.com (W.D.); qc_1009@163.com (Q.C.)

**Keywords:** dual-energy CT, ischemic stroke, endovascular thrombectomy, hemorrhagic transformation

## Abstract

Background: Dual-energy CT (DECT) shows good performance in differentiating hemorrhage from contrast staining (CS). However, no guidelines have standardized the post-endovascular thrombectomy (EVT) examination time. We evaluated the value of performing DECT immediately and 24 h post-EVT in the diagnosis and prediction of hemorrhagic transformation (HT). Methods: Two readers evaluated simulated conventional CT (sCCT) images compared with a second reading with DECT, establishing the diagnosis of HT immediately and 24 h post-EVT. Another reader’s diagnosis 2–7 days post-EVT using non-contrast CT was identified as the final diagnostic criteria. Results: DECT performed immediately and 24 h post-EVT changed 22.4% (52/232) and 12.5% (29/232) of sCCT-based HT diagnoses, respectively (χ^2^ = 10.7, *p* < 0.05). The sensitivity, negative predictive value (NPV), and accuracy of DECT performed immediately post-EVT for predicting the final diagnosis of HT were 33.6%, 58.9%, and 65.9%, respectively, whereas those for DECT performed 24 h post-EVT were 82.4%, 84.3%, and 90.9%, respectively (χ^2^ = 58.0, χ^2^ = 42.9, χ^2^ = 13.6; *p* < 0.05). The specificity and positive predictive value were both 100.0%. Delayed HT occurred in 50.0% (78/156) and 42.2% (19/45) of patients with CS diagnosed immediately and 24 h post-EVT, respectively. Conclusions: DECT performed immediately post-EVT changed a greater proportion of real-time HT diagnoses, whereas that performed 24 h post-EVT had higher sensitivity, NPV, and accuracy in predicting the final diagnosis of HT. A substantial proportion of patients with CS diagnosed at these two post-EVT timepoints subsequently developed delayed HT.

## 1. Introduction

Acute ischemic stroke (AIS) is a leading cause of disability and mortality worldwide [1]. The safety and efficacy of endovascular thrombectomy (EVT) have been confirmed in multiple randomized controlled trials, and EVT has been recommended as the first-line treatment for AIS with large artery occlusion [2,3,4]. However, post-interventional cerebral hyperdensities (PCHDs) are a common finding in non-contrast CT (NCCT) examinations after EVT due to blood–brain barrier injury. PCHDs are usually composed of contrast staining (CS) alone under the circumstance of the limited injury confined to the endothelial cell layer, whereas blood extravasation is common in the case of the degraded basal lamina [5,6,7]. As a serious complication of AIS, hemorrhagic transformation (HT) is often associated with poor prognosis or even death; hence, identifying the composition of PCHDs in the early stage after EVT is crucial [8]. Dual-energy CT (DECT) solves the problem of CS mimicking or concealing hemorrhage on conventional CT images based on the principle of three-material decomposition, which can accurately differentiate post-EVT hemorrhage from CS [9]. Currently, DECT is being increasingly used by hospitals for the early diagnosis of HT after EVT.

According to the 2019 American Heart Association/American Stroke Association (AHA/ASA) AIS guidelines, a follow-up CT or magnetic resonance imaging scan is recommended 24 h after intravenous thrombolysis before starting secondary preventive treatment with antithrombotic agents [10]. However, current guidelines still have gaps on the selection of imaging methods and timing after EVT, as well as on making post-EVT treatment decisions based on different imaging findings (HT or non-HT [CS or non-PCHD]) in clinical practice. Therefore, the time to perform DECT after EVT in patients with AIS varies between hospitals, and the two most common examination timepoints are immediately [11,12,13,14] and approximately 24 h after EVT [15,16,17,18], leading to a large heterogeneity in studies related to the clinical value of DECT and the diagnosis and treatment of post-EVT HT. For example, a recent study found that parenchymal hyperdensities on single-energy CT had a higher accuracy in predicting HT than those on virtual non-contrast images reconstructed from DECT, although the diagnostic efficacy of DECT is well established [10]. Moreover, even DECT was all performed both within 24 h after EVT, the incidence of CS could be far different between several studies, whereas CS was associated with a high incidence of delayed HT [7,16]. The results of different studies on similar questions not only confuse readers but may also indirectly affect the diagnosis, treatment, and prognosis of patients.

Few studies have compared the clinical value of DECT at different timepoints after EVT, and these studies did not investigate the predictive efficacy of DECT at different timepoints for the final diagnosis of HT [15,18]. Therefore, the primary aim of this study was to investigate how DECT performed immediately versus 24 h after EVT alters the diagnosis of HT based on conventional CT and to compare their performance in predicting the final diagnosis of HT. The secondary aim was to analyze the proportion of patients diagnosed with isolated CS and those diagnosed without HT at the two time points who subsequently developed delayed HT.

## 2. Materials and Methods

### 2.1. Patient Population

This was a retrospective study that consecutively enrolled patients with AIS who underwent EVT at Affiliated Hangzhou First People’s Hospital, Zhejiang University School of Medicine from January 2018 to November 2022. The inclusion criteria were (1) AIS was diagnosed according to the 2019 AHA/ASA AIS guidelines, indications for EVT, and received treatment, and (2) DECT examinations performed immediately (0–3 h) and 24 h [(24 ± 6) h] after EVT, and follow-up plain CT scan performed 2–7 days after EVT [14]. The exclusion criteria were (1) incomplete imaging data or the presence of artifacts affecting the diagnosis, and (2) incomplete clinical data.

This study was approved by the ethics committee of the Affiliated Hangzhou First People’s Hospital, Zhejiang University School of Medicine. (No. 2016006-01). Due to the retrospective nature of the study, the requirement for informed consent was waived. 

### 2.2. Clinical Data Extraction

Demographic and clinical information were extracted from electronic medical records retrospectively. The following variables were obtained: sex, age, comorbid medical conditions (hypertension, diabetes mellitus, atrial fibrillation, prior stroke or transient ischemia attack), smoking status, and clinical variables related to stroke diagnosis and treatment (baseline national institute of health stroke scale score; clinical positioning of stroke, i.e., anterior circulation stroke or posterior circulation stroke; intravenous thrombolysis; permanent stent placement; oral anticoagulants use; recanalization successful, i.e., the score of Thrombolysis in Cerebral Infarction scale 2b or 3; time from groin puncture to recanalization in minutes; and time from admission to recanalization in minutes).

### 2.3. Image Acquisition and Analysis

DECT was performed immediately (0–3 h) and 24 h [(24 ± 6) h] after EVT using a dual-source 128-slice CT scanner (SOMATOM Flash, Siemens Healthcare, Forchheim, Germany). The scanning parameters of DECT were as follows: 2 bulb voltages: 80 kV and Sn140 kV; reference current: 392/196 mAs; collimator width: 64 mm × 0.6 mm; automatic reconstruction layer thickness: 0.75 mm; layer spacing: 0.7 mm; scanning pitch: 0.7; rotation time: 0.5 s/r; and fusion coefficient: 0.4. The raw spiral projection data were transferred to the postprocessing workstation (Syngo. CT Dual-Energy Brain Hemorrhage; Siemens) to generate a simulated conventional CT (sCCT, equivalent to a 120-kV conventional single-energy CT) image, virtual non-contrast (VNC), and iodine overlay maps (IOM). Conventional NCCT was performed 2–7 days post-EVT to assess HT. The NCCT scanning parameters were as follows: bulb voltages: 120 kV; effective current: 340 mA; scanning pitch: 1.2; rotation time: 0.5 s/r; and layer thickness: 5 mm.

For DECT images obtained immediately and 24 h after EVT, two radiologists with >5 years of experience in neuroimaging diagnosis separately firstly reviewed the sCCT images alone and, in a second reading, these images along with the IOM and VNC images [9,15,18]. HT presence was assessed by analyzing the presence and nature of PCHDs. PCHDs were defined as areas of parenchyma hyperdense with a surface area ≥ 0.1 cm^3^ after EVT and that increased in density by at least 5 HU compared with the unaffected contralateral side [5,19]. PCHDs can be diagnosed as CS, intracranial hemorrhage (ICH), or ICH combined with CS. The classical definitions of CS and ICH on DECT have been described in detail elsewhere [9]. ICH, whether combined with CS or not, was diagnosed as the presence of HT, whereas CS or non-PCHD indicated the absence of HT. Any disagreements were resolved by consensus.

Another experienced neuroradiologist independently reviewed the follow-up NCCT to make a final diagnosis of the presence or absence of HT using the following diagnostic criteria: on re-examination of the CT images, persistent or enlarged PCHDs suggested the presence of HT, and non-HT was diagnosed if PCHDs were significantly absorbed or disappeared [19]. In addition, the definition of delayed HT in this study was consistent with the classic definition, which referred to the new detection of emerging ICH on subsequent CT scans in patients with a first DECT diagnosis of no ICH after EVT [7]. Each review by all radiologists was performed under the condition of blinding the clinical information. 

### 2.4. Statistical Analysis

Statistical analyses were performed on SPSS package (Version 25.0, IBM Corp., Armonk, NY, USA). The continuous values were presented as mean ± standard deviation or median with interquartile ranges, whereas the categorical variables were presented as number and percentage. Sensitivity, specificity, positive predictive value (PPV), negative predictive value (NPV), and accuracy were all calculated with 95% confidence intervals. The significance of differences between proportions was calculated using the chi-square test or McNemar’s test. McNemar’s test was used to compare sensitivity, specificity, and accuracy, and the chi-square test was used to compare the PPV and NPV. All tests were two-tailed, and a *p* value < 0.05 indicated a statistically significant difference. Unweighted κ-values with 95% confidence intervals were used to assess the level of inter-reader agreement.

## 3. Results

**Figure 1:**
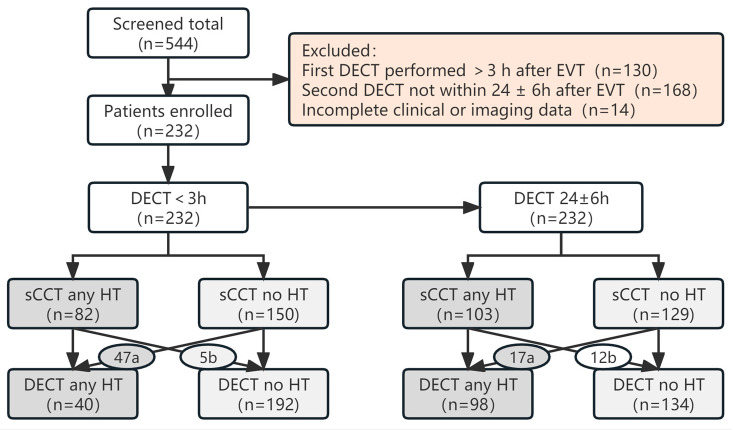
Flowchart of distribution of non-hemorrhagic transformation (non-HT) versus HT cases. a: HT was detected with Dual-energy CT (DECT) in patients with no HT on simulated conventional CT (sCCT). b: Contrast staining mimicking HT.

A total of 544 patients with AIS underwent EVT during the study period. Among these, 312 did not fulfill the inclusion criteria: 130 and 168 patients were excluded because the first DECT was performed later than 3 h after EVT and the second was not within the range of 24 ± 6 h after EVT, respectively, and another 14 patients were excluded because of incomplete clinical or imaging data. A recruitment flowchart combining the results of sCCT and DECT is shown in Figure 1. The demographic and clinical data of the 232 enrolled patients are shown in Table 1.

As shown in Table 2, of the 232 patients with AIS enrolled in this study, the findings on sCCT images immediately after EVT were 82 patients showing HT and 150 showing no HT. DECT performed in the same period changed the diagnosis to 47 with HT versus 5 without HT, i.e., the findings were 40 patients with HT and 192 without HT (consisting of 156 CS and 36 non-PCHD cases). sCCT findings obtained 24 h after EVT revealed 103 patients with HT and 129 without HT. Using DECT, the number of patients diagnosed with HT was reduced to 98, whereas that of non-HT patients increased to 134 (45 CS and 89 non-PCHD cases). In the presence or absence of HT, DECT was performed immediately and 24 h after EVT changed the sCCT-based diagnosis in 22.4% (52/232) and 12.5% (29/232) of the patients, respectively. A significant difference (χ^2^ = 10.7, *p* < 0.05) was noted in the proportion of changes in diagnostic results (i.e., error rate diagnosed using sCCT). In addition, whether immediately or 24 h after EVT, the inter-reader agreement of DECT (κ = 0.83 [95% CI 0.73–0.92], κ = 0.86 [95% CI 0.80–0.93]) was higher than that of sCCT (κ = 0.66 [95% CI 0.55–0.76], κ = 0.59 [95% CI 0.49–0.69]). 

**Figure 2:**
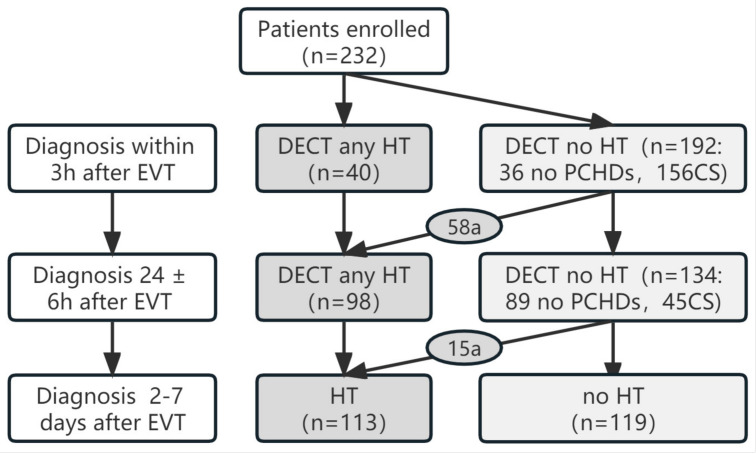
Diagnosis flowchart of delayed hemorrhagic transformation (HT) immediately and 24 h after endovascular thrombectomy (EVT). a: Delayed HT was diagnosed with reexamination in patients with no HT. CS, contrast staining; PCHDs, post-interventional cerebral hyperdensities.

Using NCCT 2–7 days after EVT, 113 HT and 119 non-HT cases were diagnosed (Table 2). The diagnosis of HT based on DECT both immediately and 24 h after EVT was correct, whereas delayed HT occurred in 73 (71 CS and 2 non-PCHD) and 15 (14 CS and 1 non-PCHD) non-HT cases diagnosed using DECT immediately and 24 h post-EVT, respectively (Figure 2). Combined with these results, the sensitivity, specificity, PPV, NPV, and accuracy of DECT performed immediately after EVT in predicting the final diagnosis of HT at 2–7 days were 33.6% (25.4–42.9%), 100% (95.9–100%), 100% (95.3–100%), 58.9% (51.5–65.8%) and 65.9% (59.6–71.2%), respectively, whereas those for DECT performed 24 h after EVT were 82.4% (74.1–88.5%), 100% (95.9–100%), 100% (89.1–100%), 84.3% (75.8–89.8%), and 90.9% (86.5–94.1%), respectively (Table 3). The sensitivity, NPV, and accuracy of DECT 24 h post-EVT were higher than those of DECT immediately after EVT (χ^2^ = 58.0, χ^2^ = 42.9, χ^2^ = 13.6; *p* < 0.05). 

**Figure 3:**
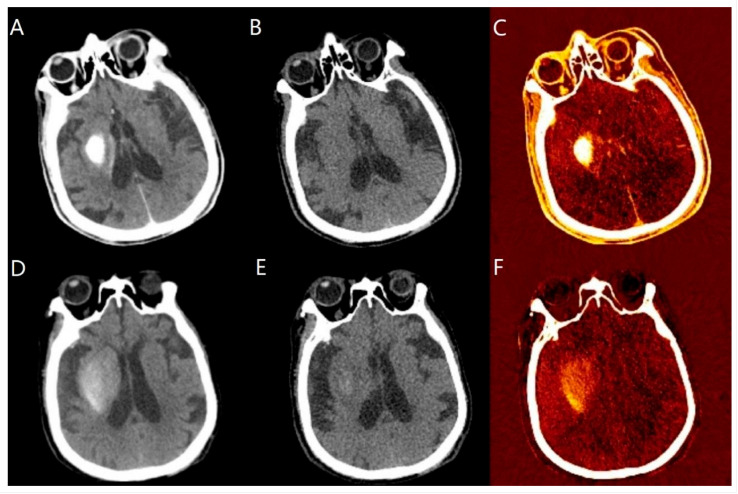
Representative case of delayed hemorrhagic transformation (HT). Immediate post-EVT DECT panel (**A**–**C**); 24-h post-EVT DECT panel (**D**–**F**). (**A**) simulated conventional CT (sCCT) image showing post-interventional cerebral hyperdensity (PCHD), (**B**) virtual non-contrast (VNC) imaging showing no PCHD, and (**C**) iodine overlay map (IOM) image showing PCHD. A diagnosis of no HT was then made at that time. (**D**–**F**) PCHD was observed on sCCT, VNC, and IOM images, and therefore delayed HT was diagnosed. DECT, Dual-energy CT; EVT, endovascular thrombectomy.

Delayed HT (a representative case is shown in Figure 3) occurred in 41.1% (79/192) of non-HT and 50.0% (78/156) of CS cases diagnosed using DECT immediately after EVT, whereas this proportion was 15.6% (21/134) and 42.2% (19/45) when diagnosed using DECT 24 h post-EVT, respectively.

## 4. Discussion

As a frontline treatment for AIS with large artery occlusion, EVT is increasingly being applied in clinical practice, and studies have shown that up to 15–20% or more of ischemic stroke patients may be eligible for EVT treatment [2]. The advancement in treatment methods also raises higher demands on post-EVT imaging examination methods. With the gradual promotion and application of DECT equipment in clinical practice and the integration of advanced technical methods, such as the three-material algorithm with DECT, early post-EVT differentiation between ICH and CS is no longer difficult. However, there are currently no authoritative guidelines providing guidance or recommendations on when to perform DECT examinations after EVT, leading to significant variations in the application of post-EVT DECT examinations across different medical centers. The current understanding of the clinical value of DECT examination at different time points after EVT is limited. Existing studies have mainly focused on the proportion of sCCT diagnosis changed by DECT at different time points after EVT, but whether this proportion varies significantly with different examination times has not been explored in depth at the statistical level [15,18]. Based on the previous classic studies, this study attempted to compare and re-examine the diagnostic and predictive value of DECT scans for HT at the two most commonly used time points (immediately and 24 h after EVT) so as to provide a basis for a more scientific and rational arrangement of the DECT examination time for patients.

In this study, compared to patients who underwent immediate post-EVT DECT, the proportion of patients diagnosed with HT using DECT 24 h after EVT increased from 17.2% (40/232) to 42.2% (98/232). Meanwhile, the detection rate of CS in patients without HT decreased from 67.2% (156/232) to 19.4% (45/232). In many previous studies in which DECT was performed immediately after EVT, the detection rate of HT was ≤34.0%, whereas that of CS was ≥37.4% [11,12,13,14,18]. In several studies using DECT 24 h after EVT, the detection rate of HT was ≥27.4%, whereas that of CS was ≤28.0% [15,16,17,18]. The detection rates of HT and CS at these two post-EVT timepoints in this study were approximately similar to those reported in previous studies, and the DECT results changed with different post-EVT examination times [11,12,13,14,18]. The later the post-EVT DECT time, the higher the detection rate of HT and the lower the detection rate of CS. Current post-EVT secondary prophylactic antithrombotic therapy usually needs to be initiated after confirming the absence of HT [20,21]. In view of the different detection rates of HT immediately and 24 h after EVT, if the diagnostic and predictive value of DECT for HT at these two post-EVT timepoints can be confirmed, this may help to develop a more reasonable clinical diagnosis and treatment strategy for patients after EVT.

In this study, compared with sCCT, DECT performed immediately, and 24 h post-EVT changed the radiologic report regarding posttreatment HT in a considerable proportion of patients undergoing EVT; immediate postoperative DECT changed the diagnosis in a larger proportion (22.4% [52/232] versus 12.5% [29/232]), and the difference was statistically significant. Almqvist et al. found that DECT performed within 18 h after EVT changed 47.7% (21/44) of HT diagnoses compared with sCCT performed at the same time, which was >10.9% (36/328) within 36 h after EVT [15]. Their findings are similar to those of this study, which concluded that earlier post-EVT DECT scans may change a greater proportion of HT diagnoses [15,18]. Due to the lack of authoritative guidelines reporting the best time to initiate antiplatelet or anticoagulant drug therapy for patients with HT after EVT, it would be more meaningful to perform DECT earlier after EVT for definitive immediate diagnosis and early initiation of possible antithrombotic therapy [10].

Using NCCT reexamination results at 2–7 days after EVT as the final diagnostic criteria, it was found that the sensitivity, NPV, and accuracy of DECT performed 24 h post-EVT for predicting HT were higher than those of DECT performed immediately post-EVT. The diagnostic or predictive efficacy of DECT for HT was often examined in previous studies at a single timepoint after EVT. Although some studies performed DECT scanning at the same timepoints after EVT, the differences in other factors, such as treatment methods, time of follow-up imaging, and final diagnostic criteria, led to significant heterogeneity among the studies [5,11,12,13,22]. Therefore, the conclusions drawn from the comparisons of these results had inevitable limitations. The consistency of factors other than the postoperative examination time was ensured when comparing the predictive efficacy of DECT for the final HT diagnostic results immediately versus 24 h after EVT in this study, making the conclusions more persuasive. A recent meta-analysis found that the post-EVT examination time was a factor that causes heterogeneity among studies on the diagnostic accuracy of DECT for HT after EVT, which further confirmed this conclusion [23]. To sum up, DECT performed immediately after EVT could identify HT and CS in the early post-EVT period, which might not only help in initiating antithrombotic therapy in the early stage but may also have excellent specificity and positive predictive value for the prediction of the final diagnosis of HT. However, 24-h post-EVT DECT could reduce the detection rate of delayed HT in patients diagnosed with non-HT and have a higher predictive efficacy for the final diagnosis. Thus, DECT immediately and 24 h after EVT have their respective advantages. It would be beneficial if a unified specification of post-EVT DECT examination timepoint could be developed, and the post-EVT examination time and frequency can be flexibly selected according to the specific clinical and patient conditions.

Currently, there are several different definitions of delayed HT in clinical practice. Some studies consider any new appearance or persistent enlargement of ICH on follow-up CT images, regardless of its presence on the initial post-EVT CT, as delayed HT [5]. Other studies exclude cases where ICH was diagnosed on the first CT examination [7,11,14]. In this study, we adopted the definition consistent with the latter approach. This is because understanding delayed HT and determining its incidence rate is essential, given the potential risks associated with this type of ICH, such as worsened patient condition, prognosis, and increased risk of death upon occurrence. Moreover, treatment strategies for EVT vary among different medical centers, with some centers leaning towards bridging therapy and others preferring direct EVT [24]. These differences in treatment modalities may influence the proportion of delayed HT in patients post-EVT, as various factors, including direct vascular damage caused by different surgical methods during interventional therapy and the toxic side effects of thrombolytic drugs, are associated with post-EVT hemorrhagic transformation. Currently, there is very limited research on the incidence of delayed HT after EVT [7]. We believe that reporting this proportion is highly meaningful and even has the potential to become an indicator for evaluating the safety and reliability of a certain treatment method. However, the proportion of delayed HT is directly related to the time of examination, and thus standardizing the examination time is crucial for determining the occurrence of delayed HT in patients initially diagnosed without ICH at different time points. While delayed HT is generally considered secondary to PCHD, this study, along with the previous literature, has demonstrated cases of delayed HT in patients without PCHD postoperatively. Therefore, this study analyzed the proportion of delayed HT both in patients initially diagnosed as no-HT after EVT and in patients with pure CS.

This study showed that the proportion of patients in whom delayed HT was observed among those without HT diagnosed using DECT decreased from 41.1% to 15.6%, moving from immediately to 24 h after EVT. Despite cases of delayed HT in non-PCHD patients at these two timepoints, the vast majority of delayed HT cases were found in patients with CS. We found that >40.0% of patients with CS diagnosed using DECT at both post-EVT timepoints developed delayed HT within 2–7 days. Previous studies found that isolated CS was associated with delayed HT; however, few studies determined the proportion of patients diagnosed with CS who subsequently developed delayed HT at any specific timepoint post-EVT, and only one study reported that 50.0% of patients with CS diagnosed 12–24 h after EVT subsequently developed delayed HT [7,16,18]. This study was novel in simultaneously reporting the detection rate of delayed HT in patients diagnosed with CS immediately and 24 h after EVT. It is true that delayed HT is not a new concept in clinical and scientific research, and we believe that these data are very meaningful and can provide reference information for other scientific research or guideline developments in the future. In summary, delayed HT occurred in a substantial proportion of patients with CS detected immediately and 24 h after EVT, and combined with the current controversy as to whether CS impacted functional outcomes or mortality, this may require more clinical attention [7,16].

This study has some limitations. First, this was a single-center, retrospective study. Although the sample size was slightly larger than that in the previous study of the same type [18], selection bias cannot be completely avoided. Second, due to the lack of stroke etiology (using the Trial of Org 10,172 in Acute Stroke Treatment [TOAST] classification) in quite a few patients, as well as preoperative antiplatelet aggregation medication use and compliance data, these variables were not included in the demographic and clinical data of this study. Further multicenter, large-sample, and well-designed prospective studies are needed in the future. Finally, the proportion of patients with AIS in whom direct EVT was performed who met the indications in our hospital was higher than that in previous studies, and the differences in treatment modalities may also have limited the extrapolation value of the conclusions of this study to some extent [24,25].

## 5. Conclusions

DECT performed both immediately and 24 h after EVT changed the diagnosis of HT in several patients with AIS, and the proportion of changes with DECT performed immediately post-EVT examination was greater. Compared with immediate post-EVT DECT, 24-h post-EVT DECT had higher sensitivity, NPV, and accuracy for predicting the final diagnosis of HT. Overall, DECT performed immediately after EVT and that performed 24 h after EVT have their own advantages and effects. In clinical practice, DECT can be performed at both of these timepoints or selectively at either one, based on the patient’s specific condition and clinical requirements. In addition, >40.0% of patients with CS diagnosed using immediate and 24-h post-EVT DECT subsequently developed delayed HT, which may require more clinical attention. This study can provide a reference basis for the development of guidelines related to the selection of imaging methods before antithrombotic and anticoagulant therapy after EVT in patients with AIS in the future.

## Figures and Tables

**Table 1:** Demographic and Clinical Data (*n* = 232).

Characteristic	Mean ± SD or Median (IQR) or *n* (%)
Female, *n* (%)	91 (39.2%)
Age (years)	69.6 ± 11.7
Comorbid conditions	
Hypertension, *n* (%)	167 (72.0%)
Diabetes mellitus, *n* (%)	32 (13.8%)
Atrial fibrillation, *n* (%)	94 (40.5%)
Prior stroke or TIA, *n* (%)	36 (15.5%)
Smoking, *n* (%)	65 (28.0%)
Clinical variables	
Baseline NIHSS score	16 (12–20)
Anterior circulation stroke, *n* (%)	198 (85.3%)
Intravenous thrombolysis, *n* (%)	34 (14.7%)
Permanent stent placement, *n* (%)	66 (28.4%)
Oral anticoagulants, *n* (%)	54 (23.3%)
TICI 2b or 3, *n* (%)	222 (95.7%)
Onset to puncture (min)	385.5 (293.0–523.8)
Puncture to reperfusion (min)	60.0 (39.0–87.8)

TIA, indicates transient ischemic attack; NIHSS, National Institutes of Health Stroke Scale; TICI, Thrombolysis in Cerebral Infarction scale score; SD, standard deviation; IQR, interquartile range.

**Table 2:** HT assessment results at different stages after EVT (*n* = 232).

	Immediate after EVT		24 h after EVT		2–7 d after EVT
	sCCT	DECT	sCCT	DECT	
No HT, *n*	150	192	129	134	119
Contrast Staining, *n*	113	156	43	45	N/A
No PCHDs, *n*	37	36	86	89	N/A
HT, *n*	82	40	103	98	113

HT, hemorrhagic transformation; EVT, endovascular thrombectomy; DECT, Dual-energy CT; sCCT, simulated conventional CT; PCHDs, post-interventional cerebral hyperdensities; N/A, not applicable.

**Table 3:** Comparison of evaluation indicators for predicting the final diagnosis of HT with immediate and 24-h DECT after EVT.

	Sensitivity (%)	Specificity (%)	PPV (%)	NPV (%)	Accuracy (%)
DECT immediately after EVT	33.6% (40/119)	100% (113/113)	100% (40/40)	58.9% (113/192)	65.9% (153/232)
DECT 24 h after EVT	82.4% (98/119)	100% (113/113)	100% (98/98)	84.3% (113/134)	90.9% (211/232)
χ^2^ value *	58.0	0	0	42.9	13.6
*p* value	<0.001	1	1	<0.001	<0.001

* McNemar’s test was used to compare sensitivity, specificity, and accuracy, and the chi-square test was used to compare the PPV and NPV. EVT, endovascular thrombectomy; DECT, Dual-energy CT; HT, hemorrhagic transformation; PPV, positive predictive value; NPV, negative predictive value.

## Data Availability

The data are available upon request from the corresponding author.
